# Older Adults Engage With Personalized Digital Coaching Programs at Rates That Exceed Those of Younger Adults

**DOI:** 10.3389/fdgth.2021.642818

**Published:** 2021-08-06

**Authors:** Sarah A. Graham, Natalie Stein, Fjori Shemaj, OraLee H. Branch, Jason Paruthi, Stephen Chad Kanick

**Affiliations:** Lark Health, Mountain View, CA, United States

**Keywords:** telemedicine, mobile health, engagement, chronic disease management, geriatric population, preventative care

## Abstract

**Background:** The US population is aging and has an expanding set of healthcare needs for the prevention and management of chronic conditions. Older adults contribute disproportionately to US healthcare costs, accounting for 34% of total healthcare expenditures in 2014 but only 15% of the population. Fully automated, digital health programs offer a scalable and cost-effective option to help manage chronic conditions. However, the literature on technology use suggests that older adults face barriers to the use of digital technologies that could limit their engagement with digital health programs. The objective of this study was to characterize the engagement of adults 65 years and older with a fully automated digital health platform called Lark Health and compare their engagement to that of adults aged 35–64 years.

**Methods:** We analyzed data from 2,169 Lark platform users across four different coaching programs (diabetes prevention, diabetes care, hypertension care, and prevention) over a 12-month period. We characterized user engagement as participation in digital coaching conversations, meals logged, and device measurements. We compared engagement metrics between older and younger adults using nonparametric bivariate analyses.

**Main Results:** Aggregate engagement across all users during the 12-month period included 1,623,178 coaching conversations, 588,436 meals logged, and 203,693 device measurements. We found that older adults were significantly more engaged with the digital platform than younger adults, evidenced by older adults participating in a larger median number of coaching conversations (514 vs. 428) and logging more meals (174 vs. 89) and device measurements (39 vs. 28) all *p* ≤ 0.01.

**Conclusions:** Older adult users of a commercially available, fully digital health platform exhibited greater engagement than younger adults. These findings suggest that despite potential barriers, older adults readily adopted digital health technologies. Fully digital health programs may present a widely scalable and cost-effective alternative to traditional telehealth models that still require costly touchpoints with human care providers.

## Highlights

- Personalized digital health programs can meet the growing needs of a rapidly expanding population of older adults.
- Older adults over 65 years showed greater engagement than adults aged 35–64 years with a fully automated health coaching platform.
- Engagement of older adults in a fully digital health platform highlights the potential for widespread adoption, and this supports continued research to optimize digital health interventions for older adult users.

## Introduction

Digital health has grown considerably in recent years, with revenue increasing from $4.4 billion in 2016 to $6 billion in 2017 and an estimated 200 new health apps being released per day (1). Since the onset of the COVID-19 pandemic in 2019, telehealth and digital health utilization rates have further increased (2, 3) with high patient satisfaction (4). The growth of digital health coincides with the US population aging, with adults 65 years and older comprising 15% of the population in 2014 and projected to grow to 21% in 2030 (5). Despite accounting for only 15% of the population, older adults accounted for 34% of total healthcare expenditures (6). Digital health innovations offer an affordable and scalable mechanism to address older adults' unique needs, helping them better manage their health and retain their autonomy (7, 8). However, to be effective for these purposes, older adults must engage with digital health technologies. A variety of digital health offerings are covered by Medicare (9, 10), but the technologies used to enable such programs may present unique barriers to older adults such as prior experience, attitudes, usability, trust, and physical and cognitive abilities (11, 12).

Older adults have a high prevalence of chronic diseases; 86% have at least one chronic condition such as diabetes or hypertension, 56% have two, and 23% have at least three (13). Self-management of these chronic conditions is essential to minimize healthcare spending. In 2016 alone, $730 billion was attributable to modifiable risk factors including high body mass index (BMI), blood pressure, and fasting glucose, and the largest fraction was for those aged 65 and older (14). Digital health programs may help older adults manage modifiable risk factors through interventions that engender positive behavior changes in physical activity, weight management, nutrition, medication adherence, and monitoring of clinical indicators like blood pressure and glucose (15, 16). Digital health programs may increase access to primary and specialty care, especially in remote or underserved areas or in populations with challenges like poor mobility (17). By increasing access to care and improving patient health, digital health programs may lessen the burden on healthcare systems (18).

Adoption and use of digital technologies by older adults are important topics, as these individuals tend to be slower than younger adults to adopt new technologies (19). However, older adults are rapidly integrating technology into their lives and are more likely to use technology when they perceive a benefit (20). Questions around older adult engagement with digital health technologies are important, as greater engagement has been associated with improved health outcomes (21, 22). Additionally, elucidating interactions between users and digital platforms helps to tailor these platforms to user preferences, which is associated with increased engagement (23). Though digital health appears promising for older adults, there are little data characterizing their engagement with digital health platforms. This knowledge gap hinders the potential for digital health programs to better serve the needs of older adults. Investigations of older adults' engagement with fully digital health platforms are necessary to help pave the way for the field and inform future studies and interventions.

The purpose of the present study was to characterize the engagement of adults 65 years and older with a mobile digital health platform called Lark Health and to compare their engagement to that of adults aged 35–64 years. We chose the comparison group of 35–64 years based upon this group having adopted digital technologies later in life, rather than having grown up with such technologies (24). The Lark digital health platform delivers personalized health coaching to promote wellness or to prevent, delay, or manage chronic diseases through promoting positive behavior changes. Lark programs are delivered *via* artificial intelligence (AI) with a responsive coaching interface on a smartphone. We analyzed data from users enrolled across four digital health programs to determine whether engagement, defined as participation in coaching conversations, meal logging, and device measurements, varied by age. We hypothesized that older adults would have less engagement in the digital platform than younger adults due to barriers to technology use common to this age group, which would be reflected by lower participation in coaching conversations and fewer meals logged and device measurements.

## Methods

### Study Design

This was a longitudinal, observational study of participants who were users of the Lark Health disease management and prevention programs. We considered measures of engagement from the program start until 12 months later. The study received exemption status from Advarra (Protocol #Pro00047181) Institutional Review Board (IRB) for retrospective analyses of previously collected and de-identified data.

### Participants and Recruitment

**Figure 1:**
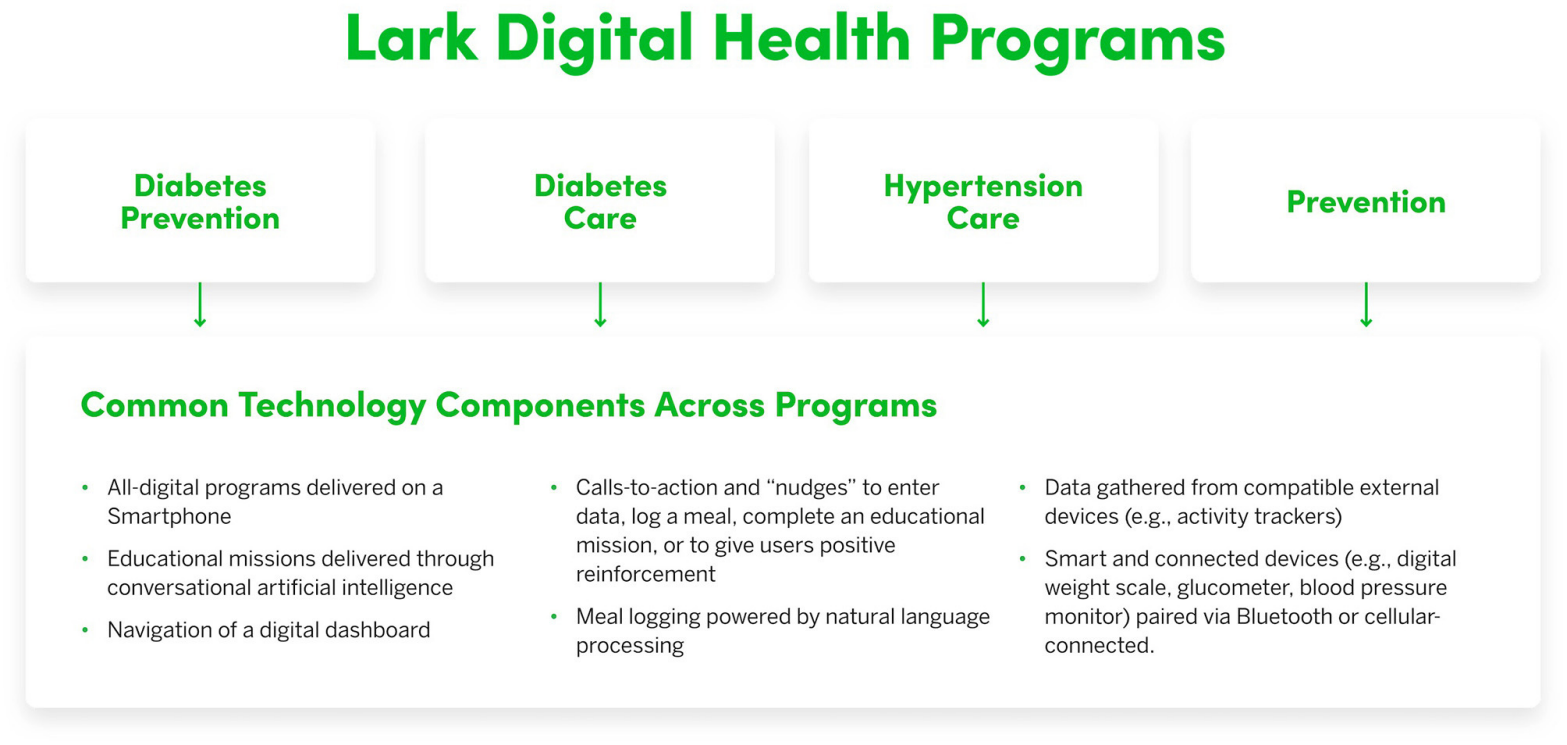
Overview of the common technological components of the Lark digital health programs.

Participants in this study were individuals who qualified for any of four digital health programs (see Figure 1) offered through existing partnerships between Lark Health and health insurance companies, employers, or other organizations, and who owned an Android-enabled smartphone or iPhone.

Lark recruits eligible users *via* direct referrals from health plans and/or healthcare providers, digital awareness campaigns (e.g., Facebook ads), and a large managed services organization. The Lark programs are a covered service under the insurance plans of these users. Eligibility differs for each clinical program based on the program focus [e.g., Diabetes Prevention Program users must meet risk criteria established by the Centers for Disease Control and Prevention (25), Diabetes Program users must have a diagnosis of diabetes, and Hypertension Program users must have a diagnosis of hypertension]. A user's health plan confirms their eligibility for a particular program prior to enrollment. There are no specific eligibility requirements for the General Wellness Program, and these users are eligible to enroll if their insurance covers preventive wellness programs. Participants who opted in a program received a link *via* text message to download the Lark program to their smartphones. Some participants received a connected device (e.g., digital weight scale) as a part of their specific program.

Participant inclusion criteria were: (1) enrollment in a Lark Health program between January 1st, 2019 and July 28th, 2019; (2) aged 35 years and older; (3) those who received a connected device as a part of their program; and (4) those who completed at least one educational mission (i.e., an educational lesson that included a series of automated check-ins with the digital coach and coaching conversations around a topic related to the program focus). We selected the date-range criteria to reduce time-dependent variations in the content of coaching and types of participant-coach interactions offered within each program. We further focused our analyses by excluding young adults 18–34 years who would require separate considerations due to lifetime technology exposure, users who did not have a connected device since they were not participating in a full version of a program, and users who did not complete any educational missions since they did not demonstrate a minimum level of intent to participate in their program.

### Digital Health Platform

The Lark digital health platform provides automated and personalized coaching using conversational AI. Each program has weekly “educational missions” consisting of daily check-ins and educational material around a weekly topic related to the focus of each program. The Lark programs differ in clinical focus and content (e.g., diabetes prevention, hypertension management) and employ a standard set of engagement methods that include automated coaching conversations, meal logging, and device measurements. Every Lark program includes AI coaching on lifestyle choices such as healthy eating, physical activity, sleep, and stress management. The AI coach employs elements of cognitive behavioral therapy to encourage users to adopt healthy behaviors and build self-management skills and knowledge to sustain these behaviors. Users receive regular “calls-to-action” and “nudges” that either encourage them to engage with the Lark platform through actions like having a coaching conversation or offer them positive reinforcement (e.g., great job on your walk today). Users have the option to set a weight-loss goal and receive personalized coaching. Lark responds immediately with personalized feedback when users log data such as weight or meals, or when they indicate they want to have a conversation. Lark also provides daily and weekly summaries of progress. The intuitive meal-logging system uses natural language processing to provide personalized coaching regarding meal content and quality. Lark can also gather data from external devices like activity trackers that are connected to Google Fit or iOS Health Kit. The AI coach is available for unlimited use 24 h a day if users want to check in to discuss challenges or progress. The main technological aspects of these programs are summarized in Figure 1.

### Measures of Engagement With the Digital Health Platform

We defined three metrics to quantify the engagement of users with the digital health platform. *Coaching conversations* included interactions between the AI coach and user and included educational missions. *Meals logged* included data provided by users regarding food intake. *Device measurements* included measurements obtained from the smart and connected devices (i.e., digital weight scale, glucometer, or blood pressure monitor).

We considered the total number of coaching conversations, meals logged, and device measurements experienced over the first 12 months after the program start date. We did not separately assess engagement metrics per program due to uneven sample sizes, but we did separately analyze two program-specific groupings, (1) clinically oriented (diabetes prevention, diabetes care, and hypertension care) and (2) wellness (prevention).

### Statistical Analyses

We conducted all statistical tests in Python version 3.7.3. We checked distributions for each variable and compared age groups on continuous measures with the Mann-Whitney *U*-test (*U*-statistic) using the “mannwhitneyu” function from the scipy.stats module in python due to non-normal data, and Chi-Square tests (χ^2^ statistic) using the “chi2_contingency” function from scipy.stats for categorical data. Users self-reported their age, gender, weight, and height upon enrollment in the Lark digital platform. We calculated body mass index (BMI; kg/m^2^) from height and weight. We reported medians with interquartile (IQ) ranges for demographics and user characteristics for: (1) all users, (2) users 65 years and older, and (3) users 35 to 64 years, as well as the distribution of users across program types (Table 1).

**Table 1:** Participant demographics and characteristics and distribution of users across programs.

		**Full sample (*N* = 2,169)**	**35–64 years (*n* = 1,868)**	**65+ years (*n* = 301)**	
		**Median [IQ range]**	**Median [IQ range]**	**Median [IQ Range]**	***U*-Stat.; *p*-val**
**Age**	[years]	53 [45, 60]	51 [43, 57]	68 [66, 71]	0; *p* < 0.01
**Weight**	[kg]	92 [79, 108]	93 [79, 109]	87 [75, 101]	199,447; *p* < 0.01
**Height**	[cm]	168 [163, 175]	168 [163, 175]	168 [160, 175]	255,768; *p* = 0.01
**BMI**	[kg/m^2^]	32 [29, 36]	32 [29, 37]	31 [28, 35]	174,003; *p* < 0.01
		***N*** **[%]**	***n*** **[%]**	***n*** **[%]**	**χ**^**2**^ **Stat.;** ***p*** **-val**
**Gender**	F	1,448 [67]	1,264 [68]	184 [61]	11; *p* < 0.01
	M	708 [33]	596 [32]	112 [37]	
	N/A	13 [0]	8 [0]	5 [2]	
**Race**	White	1,570 [72]	1,326 [71]	257 [85]	11; *p* = *0.001*
	Not White	599 [28]	542 [29]	44 [15]	
**Ethnicity**	Hispanic or Latino	210 [10]	192 [10]	13 [4]	27; *p ≤ 0.0001*
	Not Hispanic or Latino	1,959 [90]	1,676 [90]	288 [96]	
		***N*** **[%]**	***n*** **[%]**	***n*** **[%]**	**χ**^**2**^ **Stat.;** ***p*****-val**
**Programs**	Diabetes prevention	1,396 [64]	1,201 [64]	195 [65]	3; *p* = 0.49
	Diabetes care	86 [4]	69 [4]	17 [6]	
	Hypertension care	151 [7]	130 [7]	21 [7]	
	Prevention	536 [25]	468 [25]	68 [22]	

*Data presented for the full sample and stratified by age into older (65+ years) and younger (35–64 years) groups. Statistical comparisons are between age groups for demographics, characteristics, and distribution of users across programs*.

We compared age groups (i.e., older adults vs. younger adults) on engagement metrics (i.e., number of coaching conversations, meals logged, and device measurements). We also compared these engagement metrics between age groups for two program-specific groupings (i.e., clinically oriented vs. wellness) and within each age group between these program groupings. We reported both medians with IQ ranges and means with 95% CIs for all engagement metrics in Tables 2, 3. We used an alpha ≤ 0.05 to evaluate significance for all tests.

**Table 2:** Engagement metrics of users across all programs.

**Engagement metrics**	**Values**	**Full sample (*N* = 2,169)**	**35–64 years (*n* = 1,868)**	**65± years (*n* = 301)**
**Number of coaching conversations**	Median [IQ range]	437 [281, 615]	428 [276, 598]	514 [312, 720]^**^
	Mean [95% CI]	486 [474, 499]	474 [461, 488]	561 [524, 597]
**Number of meals logged**	Median [IQ range]	96 [39, 220]	89 [38, 201]	174 [54, 398]^**^
	Mean [95% CI]	176 [167, 186]	161 [151, 170]	273 [240, 306]
**Number of device measurements**	Median [IQ range]	30 [10, 70]	28 [10, 67]	39 [15, 101]^**^
	Mean [95% CI]	71 [55, 87]	69 [51, 88]	82 [69, 94]

Statistical significance based on comparison of the medians between age groups and denoted by ^*^p ≤ 0.05;

** *p ≤ 0.01 next to older adult medians*.

*Data presented for the full sample and stratified by age into older (65+ years) and younger (35–64 years) groups. Median [Interquartile (IQ) Range] and Mean [95% Confidence Interval (CI)] provided for all comparisons*.

**Table 3:** Engagement metrics of users over a 12-month period broken down by program-specific grouping into clinically oriented (diabetes prevention, diabetes care, and hypertension care) and wellness (prevention).

**Clinically oriented programs**				
**Engagement metrics**	**Values**	**Full sample (*n* = 1,633)**	**35–64 years (*n* = 1,400)**	**65± years (*n* = 233)**
Number of coaching conversations	Median [IQ Range]	437 [281, 624]	431 [278, 614]	485 [300, 706]^**^
	Mean [95% CI]	494 [479, 509]	486 [470, 503]	540 [500, 581]
Number of meals logged	Median [IQ Range]	106 [45, 224]	102 [45, 214]	141 [49, 368]^**^
	Mean [95% CI]	183 [172, 194]	171 [160, 182]	252 [215, 288]
Number of device measurements	Median [IQ Range]	30 [9, 71]	28 [9, 67]	38 [13, 112]^**^
	Mean [95% CI]	75 [54, 96]	73 [49, 97]	84 [69, 99]
**Wellness program**				
**Engagement Metrics**	**Values**	**Full Sample (*****n*** **=** **536)**	**35–64 years (*****n*** **=** **468)**	**65±** **years (*****n*** **=** **68)**
Number of coaching conversations	Median [IQ Range]	434 [284, 585]	423 [269, 561]^†^	584 [398, 767]^**^^†^
	Mean [95% CI]	463 [441, 485]	439 [417, 460]	630 [548, 712]
Number of meals logged	Median [IQ Range]	69 [27, 187]	62 [25, 152]^‡^	285 [114, 493]^**^^†^
	Mean [95% CI]	157 [139, 175]	130 [113, 147]	347 [278, 416]
Number of device measurements	Median [IQ Range]	30 [13, 68]	28 [12, 63]	40 [29, 92]^**^
	Mean [95% CI]	60 [53, 67]	58 [50, 66]	73 [53, 93]

Statistical comparisons based first on the medians between age groups and denoted by ^*^p ≤ 0.05;

** *p ≤ 0.01 next to older adult medians for both program-specific groupings. Statistical comparisons also presented based on the medians between program-specific groupings within each age group and denoted by*

† *p ≤ 0.05;*

‡ *p ≤ 0.01 next to each age-group's median under the wellness program results*.

*Data presented for the full sample and stratified by age into older (65+ years) and younger (35–64 years) groups. Median [Interquartile (IQ) Range] and Mean [95% Confidence Interval (CI)] provided for all comparisons*.

## Results

Per our inclusion/exclusion criteria, the final sample size included in the analyses was 2,169 users. Older adults aged 65+ years comprised 14% of the sample, and the remaining 86% consisted of adults aged 35–64 years. Per design, we had complete separation between age groups (Table 1). Older users were more likely to be male than younger users (37 vs. 32%; *p* < 0.01) and had a lower body weight (87 kg vs. 93 kg; *p* < 0.01) and BMI (32 vs. 33 kg/m^2^; *p* < 0.01) at program enrollment (Table 1). We did not observe a difference in the distribution of users across programs between the two age groups (*p* = 0.49).

Aggregate engagement across all users during the 12-month period included 1,623,178 coaching interactions, 588,436 meals logged, and 203,693 device measurements. We observed that older adults engaged with the Lark digital health platform to a greater degree than younger adults, evidenced by a significantly larger median number of coaching conversations (*U* = 233,794; *p* ≤ 0.01), meals logged (*U* = 212,673; *p* ≤ 0.01), and device measurements (*U* = 238,056; *p* ≤ 0.01) across all programs (Table 2).

When we separately considered user engagement per thematic program grouping (i.e., clinically oriented vs. wellness), we again observed that older adults engaged with the Lark platform to a greater degree than younger adults. Compared to younger adults, older adults had a higher median number of coaching conversations (*U* = 144,761; *p* ≤ 0.01), meals logged (*U* = 138,824; *p* ≤ 0.01), and device measurements (*U* = 140,316; *p* ≤ 0.01) for clinically oriented programs and a higher median number of coaching conversations (*U* = 10,114; *p* ≤ 0.01), meals logged (*U* = 7,909; *p* ≤ 0.01), and device measurements (*U* = 12,590; *p* ≤ 0.01) for the wellness program (Table 3).

We further found that within each age group, older adults enrolled in the wellness program had a higher median number of coaching conversations (*U* = 309,663; *p* = 0.03) and meals logged (*U* = 258,785; *p* ≤ 0.01) compared with older adults enrolled in the clinically oriented programs (Table 3). Older adults did not differ in device measurements (*p* = 0.15) between program-specific groupings. In contrast, younger adults enrolled in the wellness program had a lower median number of coaching conversations (*U* = 6,599; *p* = 0.02) and meal logging (*U* = 6,109; *p* ≤ 0.01) compared to younger adults enrolled in the clinically oriented programs (Table 3). Younger adults also did not differ in device measurements (*p* = 0.25) between program-specific groupings.

## Discussion

The present study characterized the engagement of older adults aged 65 and older with a digital health platform compared to adults aged 35–64 years. Users of the Lark digital health platform engaged with multiple modes of technology over a 12-month period, including navigating a mobile application on a smartphone, engaging in conversational AI with a digital coach, receiving and responding to prompts to interact with the platform, logging meals, and monitoring progress *via* measurements of weight, glucose, and blood pressure collected *via* smart and connected devices. Contrary to our main hypothesis, we observed that older adults engaged more with these technologies than younger adults, evidenced by engagement in a larger number of coaching conversations and more meals logged and device measurements.

### Older Adults Engaged With Fully Digital Health Programs

The higher engagement observed in older adults in this study is promising. Although the literature on the use of digital health technologies among older adults is sparse, some evidence has suggested they have lower levels of engagement than younger adults due to barriers to use (11, 12). For example, older adults experience declining physical and cognitive functioning (26), which may directly affect their ability to visually navigate a digital screen, remember how to interact with digital programs, or understand technological prompts or notifications. However, we observed that older adults engaged in more coaching conversations, logged more meals, and recorded more device measurements than younger adults. These interactions suggest that despite potential barriers, adults over 65 years of age were able to engage with an all-digital, app-based coaching platform. If we consider our results in the context of commonly cited barriers to technology use of older adults, our findings indicate that older adult users were able to optically interpret text, use touch-based interactions, navigate the in-app menu, take measurements with smart and connected digital devices, pair connected Bluetooth devices with their mobile phones, and maintain battery charge to support device use.

Trust is another commonly cited barrier to technology use of older adults, with an unwillingness to adopt technologies stemming from high perceptions of risk and desire for privacy (27). However, older adult users in this study shared personal details including their age, gender, weight, height, meal information, and health-related measurements. Potential trust-building factors that may have been uniquely appealing to older adults warrant further exploration. Research has shown that there are both enablers to trust (e.g., fair data access, ease of use, lack of judgment) and impediments (e.g., fear of data exploitation, insufficient training) that digital health services must consider when designing their platforms (28). Such elements are critical since not just adoption of, but also effective engagement with, digital health platforms is necessary to reap the greatest health benefits and sustain these benefits (29).

Studies of digital health technology use have shown that one reason why older adults may engage less with these technologies is simply that they are less likely than younger age groups to be offered digital health access by their healthcare provider (30). In fact, of those with access, one study of Canadian older adults found that older adults sustained their use of health-related mobile apps for longer than the general population (31). Older adults may be characterized in their use of digital technologies along a spectrum from non-users to savvy users like the general population (32). Despite suggestions to the contrary, some research has shown that older adults are willing to engage with new technologies and demonstrate positive attitudes toward technology (33). Although we did not independently assess each potential barrier, our results also collectively suggest that older adults will engage with digital health technologies when provided the opportunity.

### Facilitators and Patterns of Use of Fully Digital Health Programs by Older Adults

The engagement of older adults with digital health is important to the field of chronic disease management. Many chronic diseases are preventable or effectively managed through lifestyle changes (34, 35). However, direct contact with healthcare professionals that may offer conventional lifestyle behavior coaching is a challenge due to the shortage of practitioners that provide care (36), and the costs associated with regular human-provided care (37), resulting in unmet care needs of older adults. Telehealth initiatives have been successfully deployed to older adults for chronic disease management (38); however, classical models of telehealth still require costly touchpoints with human care providers to facilitate program engagement (39). The engagement of older adults in the fully digital programs assessed in this study demonstrates that older adults readily adopted programs that did not require any human touchpoints and that fully digital programs may therefore present a widely scalable and cost-effective alternative to traditional forms of telehealth.

Given that increased engagement with lifestyle interventions has been associated with improved health outcomes (40), our findings require further exploration of the underlying facilitators supporting the engagement of older adult users. There are other potential facilitators of engagement in digital health programs besides age, such as clinician referral, incentives (e.g., compensation) for participation, and disease diagnosis (41). Exploring interactions between age and other potential facilitators is an important future area of focus to determine how to best facilitate program engagement for various subgroups. We found that older and younger adults differed in their engagement patterns between wellness vs. clinically oriented programs. In younger adults, there were more coaching conversations and meals logged in the clinically oriented programs when compared to those enrolled in the wellness program. In contrast, older adults in the wellness program had more coaching conversations and meals logged when compared with older adults enrolled in the clinically oriented programs. It is possible that the digital coach presented older adults with an opportunity for social interactions that they desired (42, 43), particularly when the program was not focused on clinical issues. If older adults viewed the digital coach as a form of social support, this could explain the greater number of interactions of apparently healthy older adults enrolled in the wellness program, and this would support the use of fully digital health platforms for not only disease management but also for prevention of chronic disease—a minority focus of currently available digital platforms (44). Prevention is a critical area of focus for digital health programs because these technologies have the potential to stabilize or reverse the declining health of older adults before they need clinical intervention. Future work is necessary to elucidate these findings.

The results of this study must be considered with respect to the median age of the sample (68 years). Although our hypothesis was that older adults over 65 years would have less engagement with digital programs due to real or perceived barriers to technology use, a potential counter-hypothesis could be that the “younger” end of the older-adult spectrum may include newly retired individuals who have more free time to engage with digital technologies than working-age adults. We may have observed different results had we included the “oldest old” (≥80 years) who are even more likely to experience barriers to technology use (45).

### Strengths and Limitations

We did not directly assess health outcomes as they related to engagement metrics. Such an assessment would be complicated due to the different outcomes associated with each of the different programs and was beyond the scope of this study. However, the high level of engagement of older adults is a promising indicator of the potential for fully digital health interventions, since we know from the published literature that those who engage in lifestyle interventions to a greater degree are generally more successful (40, 46). The present study included only users of an existing commercial digital health product. However, a strength is that these participants represented real-world users of digital health programs rather than participants recruited as a part of a carefully controlled research study. Users had no contact with research staff; thus, their engagement with the digital platform can be attributed to their personal choices rather than instructions to behave in a particular manner. We observed less diversity in race and ethnicity in older adults than younger adults. Race and ethnicity have been found to be predictive of digital health and technology use, with minority populations less likely to engage (47). The fact that we had few older adult users of non-white and Hispanic/Latino origins may support these findings, and more work is necessary to improve inclusivity and help mitigate health disparities. Finally, our measures of engagement assessed the total number of engagement metrics rather than temporal patterns of user-coach interactions, which recent studies have suggested may be predictive of individual outcomes (48). A more detailed understanding of the ways in which older adult users interact with the digital platform will be key to optimizing the mechanisms of coaching delivery (e.g., content, timing, and frequency) and platform navigations.

## Conclusions

The present study found that older adults had greater engagement in coaching conversations, meals logged, and device measurements than younger adults, suggesting that older adults were able to navigate a digital screen, interact with a fully automated digital coach, and take measurements with smart and connected digital devices. Health-related digital technologies and digital coaches may offer older adults a way to manage the large amount of information associated with lifestyle behavior changes, and further, provide 24-h continuous encouragement and support in sustaining these lifestyle changes. Our findings collectively suggest that older adults will engage with digital health technologies when provided the opportunity. These findings support the use of fully digital health programs to deliver behavior change interventions for older adults and provide a foundation for future studies to explore age-specific relationships of patterns of engagement and outcomes.

## Data Availability Statement

The raw data supporting the conclusions of this article will be made available by the authors, without undue reservation.

## Ethics Statement

The studies involving human participants were reviewed and approved by Advarra Institutional Review Board approval (Protocol #Pro00047181) for retrospective analyses of previously collected and de-identified data. Written informed consent for participation was not required for this study in accordance with the national legislation and the institutional requirements.

## Author Contributions

SG, NS, and OB wrote and edited the manuscript. FS performed data analyses and edited the manuscript. JP provided clinical oversight and edited the manuscript. SK designed the study and contributed to writing and editing the manuscript. All authors contributed to the article and approved the submitted version.

## Conflict of Interest

SG, FS, NS, OB, JP, and SK receive a salary from Lark and some authors might have some stock options in the event that the company could go public at an unanticipated date in the future. The funder (Lark Technologies, Inc.) had the following involvement in the study: collection of data, analyses, and writing.

## Publisher's Note

All claims expressed in this article are solely those of the authors and do not necessarily represent those of their affiliated organizations, or those of the publisher, the editors and the reviewers. Any product that may be evaluated in this article, or claim that may be made by its manufacturer, is not guaranteed or endorsed by the publisher.
